# Unveiling two-dimensional magnesium hydride as a hydrogen storage material *via* a generative adversarial network†

**DOI:** 10.1039/d1na00862e

**Published:** 2022-04-08

**Authors:** Junho Lee, Dongchul Sung, You Kyoung Chung, Seon Bin Song, Joonsuk Huh

**Affiliations:** Department of Chemistry, Sungkyunkwan University Suwon 16419 Korea; SKKU Advanced Institute of Nanotechnology (SAINT), Sungkyunkwan University Suwon 16419 Korea; Department of Physics, Graphene Research Institute, GRI-TPC International Research Center, Sejong University Seoul 05006 Korea; Institute of Quantum Biophysics, Sungkyunkwan University Suwon 16419 Korea joonsukhuh@gmail.com

## Abstract

This study used an artificial intelligence (AI)-based crystal inverse-design approach to investigate the new phase of two-dimensional (2D) pristine magnesium hydride (Mg_*x*_H_*y*_) sheets and verify their availability as a hydrogen storage medium. A 2D binary phase diagram for the generated crystal images was constructed, which was used to identify significant 2D crystal structures. Then, the electronic and dynamic properties of the Mg_*x*_H_*y*_ sheets in low-energy periodic phases were identified *via* density functional theory (DFT) calculations; this revealed a previously unknown phase of 2D MgH_2_ with a *P*4̄*m*2 space group. In the proposed structure, the adsorption behaviors of the Li-decorated system were investigated for multiple hydrogen molecules. It was confirmed that Li-decorated MgH_2_ has an expected theoretical gravimetric density of 6 wt%, with an average H_2_ adsorption energy of −0.105 eV. Therefore, it is anticipated that *P*4̄*m*2 MgH_2_ sheets can be employed effectively as a medium for hydrogen storage. Additionally, this finding indicates that a deep learning-based approach is beneficial for exploring unrevealed 2D materials.

## Abstract 2

A two-dimensional (2D) *P*4̄*m*2 MgH_2_ sheet was unveiled with a generative adversarial network. Also, the hydrogen storage properties of Li-decorated 2D MgH_2_ were confirmed using the DFT calculations.

## Graphical abstract

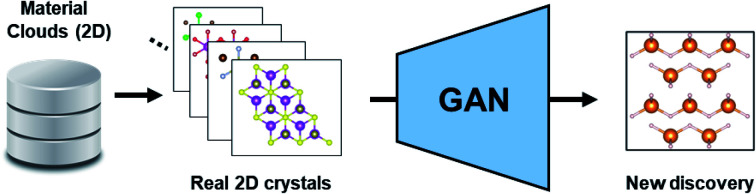

## 1 Introduction

Hydrogen energy is a promising clean energy source that can be utilized as a primary energy carrier with extensive applications.^1,2^ However, designing a crystalline material for hydrogen storage with the required high gravimetric densities for operation under ambient thermodynamic conditions remains a challenge. Consequently, numerous chemical hydrogen host materials with high hydrogen capacities have been suggested for such applications, including metal hydrides (MHs),^3,4^ chemical hydrides,^5^ and complex hydrides.^6,7^ Due to their specific properties of high thermal stability, low cost, reaction reversibility, and significant gravimetric hydrogen storage capacity, MHs have long been regarded as a promising material for hydrogen storage. However, they are limited by high desorption temperatures, low plateau pressure under ambient conditions, and relatively slow absorption/desorption kinetics; therefore, much research, including simulations, has been focused on enhancing the properties of MHs to enable their use in hydrogen storage applications.^8^

Another promising approach is the use of lightweight physical adsorbents with high surface areas, reversible H_2_ charging (and discharging), and facile kinetics, such as carbon-based nanostructures (*e.g.* nanotubes^9,10^), metal–organic frameworks,^11,12^ and covalent organic frameworks.^13,14^ Unlike chemical storage, generally, these physical adsorbents are limited by weak van der Waals interactions between adsorbed hydrogen molecules and substrates, which significantly reduces the H_2_ storage capacity of these substances due to the desorption of H_2_ at very low temperatures. Consequently, metal ions,^15,16^ including alkali metals, alkali earth metals, and transition metals, have been extensively used as dopants to produce ideal H_2_ binding strengths (∼−15 kJ mol^−1^) and increase H_2_ storage capacities.^17^ In this regard, much research has been conducted on the use of two-dimensional (2D) materials with a lightweight alkali metal or alkali earth metals for storing hydrogen. For example, many potential hydrogen storage systems have been proposed using Li-, Na-, or Ca-decorated graphene,^18–22^ boron–nitride layers,^23–25^ and carbon–nitride compounds.^26^

Recently, much research has been conducted on the structure of 2D groups of MHs, and over 100 stable MH monolayers have been reported. These 2D MHs were explored using substitution-based systematic structural investigations. They generate known layered lattice structures by combining hydrogen with all the metals in the periodic table and offer a rich array of electronic properties for use in materials ranging from metals to wide-gap semiconductors.^27^ However, despite their high gravimetric hydrogen capacity in the bulk state and their large surface area for the attachment of additional hydrogen molecules, the use of MH sheets as a hydrogen storage material has not been proposed. To date, the identification of novel 2D MHs has been *via* substitution-based methods, although depending on the combination of crystal structural motifs and atomic elements, these approaches could omit a structure from the search area.

Considerable efforts have been made to use artificial intelligence (AI) technology to predict new material structures. Compared with existing well-known structure-prediction techniques, such as evolutionary algorithms or substitution-based methods, deep learning-based approaches allow the computational cost to be reduced by exploring scoped target chemical spaces, which are represented as a continuous latent vector. In chemistry, two of the most popular generative models are the variational autoencoder and the generative adversarial network (GAN); these are used with various chemical representations, such as graph-based encoding for crystal structures, three-dimensional (3D) grid images, and point cloud images. Recently, Xie *et al.* developed a CDVAE model to design the periodic structure of stable materials.^28^ Long *et al.* developed a GAN-based inverse design framework for crystal structure prediction in the binary Bi–Se system.^29^ Fung *et al.* proposed an inverse design framework MatDesINNe and applied it to MoS_2_ for band gap engineering.^30^ A compositional conditional GAN (CCCGAN) model for designing Mg–Mn–O ternary materials was devised that successfully predicted 23 new crystal structures.^31^ We utilized this model in our study, noting that it can generate the structural motifs of materials using point-cloud-based structural image representation without any elemental consideration.This study used a deep learning-based generative model and a systematic theoretical study using the density functional theory (DFT) to propose a new crystal structure for a 2D Mg_*x*_H_*y*_ sheet. In terms of hydrogen storage, 2D MgH_2_ with a *P*4̄*m*2 space group was identified as a promising medium, and pristine and Li-doped 2D MgH_2_ were investigated as potential materials for storing hydrogen. Additionally, the adsorption energy and configuration of gas molecules were analyzed, and the adsorption conditions of multiple hydrogen molecules on Li-decorated MgH_2_ were investigated.

## 2 Computational methodology

### 2.1 DFT calculations

The calculations in this study were conducted using the DFT method with spin polarization, as implemented in the Vienna *Ab initio* Simulation Package (VASP).^32–34^ Projector-augmented wave (PAW)^35^ potentials (as parameterized by using the Perdew–Burke–Ernzerhof (PBE)^36^ functional within the generalized gradient approximation) were used together with dispersion corrections obtained from Grimme's DFT-D3 method^37^ to capture the inherent long-range atomic and molecular interactions between MH and Li, and hydrogen molecules with the Li-MH system. All calculations used a cut-off energy of 520 eV, and the system was relaxed until the forces on each atom were below 0.01 eV Å^−1^. Supercells of 2 × 2 or 3 × 3 were used with a *Γ*-centered 12 × 12 × 1 or 8 × 8 × 1 *k*-point mesh. To prevent artificial interactions, the periodic supercell was separated in the *z*-direction by a vacuum space of over 20 Å, whereas the thermal stability of the metal hydride was verified using *Ab Initio* molecular dynamics (AIMD) simulations. The molecular dynamics simulations of the MgH_2_ sheet were conducted at room temperature (300 K), and this aspect was explored using a 5 × 5 supercell. The time step was set to 3 fs, and the simulation went up to 6 ps. The ionic temperature was controlled *via* a Nosé–Hoover thermostat.

### 2.2 Generative model

For the application of deep learning techniques for exploring materials, this study used a GAN model as the learning model for its 2D crystal structure system. A GAN comprises two network groups—a generator and discriminator—which learn in a hostile manner. The goal of the generator group is to learn the distribution of training data and create a real fake image, which it does by using the Gaussian noise vector as an input to produce a fake image. The purpose of the other GAN network—the discriminator—is to learn better discrimination between the real and the fake, and it is trained to provide improved feedback to the generator. The fake outputs produced by the generator become closer to the actual image by reducing the Wasserstein distance between the distributions of fake generation and real training data. To ensure learning stability, deconvolution layers were applied to both the generator and discriminator. The GAN model for applying point cloud input to the crystal structure was obtained from Kim *et al.*,^31^ and the compositional conditional part of the model was removed and utilized for successive training progression. The deep learning model was constructed using PyTorch 1.5.

## 3 Results and discussion

### 3.1 Training of the generative model and material prediction

Before configuring a deep learning neural network for the generation of a new 2D crystal structure, it was necessary to select an appropriate representation that preserves the structural information of crystals with a reasonably sized memory. To encode the solid crystal structures into low-dimensional representations, a point-cloud-like image representation of a 2D matrix was used comprising a unit cell, lattice parameters, and fractional coordinates of each element.^31^ No additional processes were required since this representation is both invertible and consistent with the material structures. This representation is advantageous over graphical representations, as the inverse transformation from an image to a crystal structure is a precise correlation. Furthermore, this representation has low memory requirements compared with 3D voxel representations.^31^

**Fig. 1:**
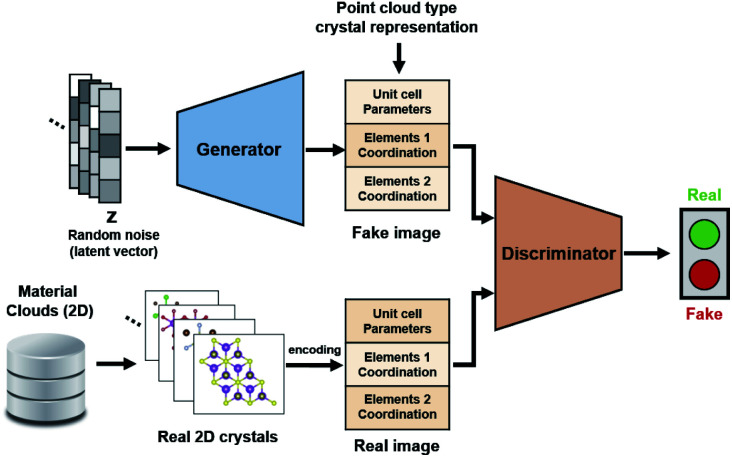
Deep learning-based generative model for the 2D crystal system used in this study.

First, 2D structural data were obtained from the Material Clouds 2D structure database,^38^ which is a data subset of approximately 1000 easily exfoliable 2D monolayers containing less than 8 atoms per unit cell. There were 134 binary and 105 ternary system nanosheets whose stability had been confirmed previously *via* DFT computational exfoliation calculations. As the aim was to generate binary MHs, the initial training set contained 134 unique structures. The problems of data shortage and invariance were addressed by augmenting the data using transitional and rotational transformations for each initial original structure of about 1000 structures.^31^ Following data preprocessing, 134 000 training data sets were available for the training of this study's model. Fig. 1 shows the complete configuration of the model.

**Fig. 2:**
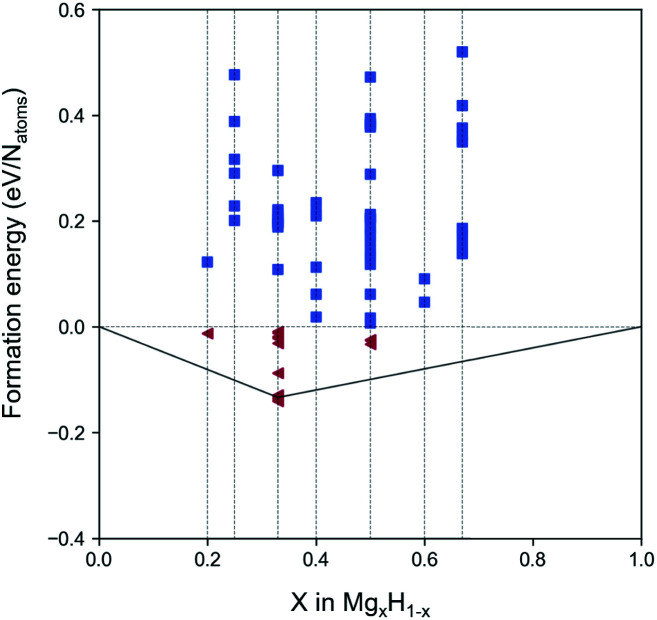
Phase diagram of the 2D Mg_*x*_H_1−*x*_ among the generated structures. The triangles colored red are the structures with negative formation energies.

The model was trained for approximately 200 epochs, and following the training with the 2D binary system data, it was confirmed from the model's losses that its learning had progressed satisfactorily. A total of 500 crystal images of binary Mg–H 2D nanosheets were generated *via* the trained generator. To evaluate the performance of the generative model, we calculated the validity and reproduction ratio. Validity is a relatively weak criterion based on simple physical rules and was proposed by Court *et al.*^39^ According to this criterion, a structure is valid as long as the shortest distance between any pair of atoms is larger than 0.5 Å. In our model, 96.8% of structures were observed as valid structures. The reproduction ratio means how much percent of the training data was reproduced during the generation. We use StructureMatcher from pymatgen,^40^ which finds the best match between two structures considering all invariances of materials. The matching criterion parameter is set as stol = 0.5, angle_tol = 10, and ltol = 0.3. Totally, 87.3% of the structural motifs in the training data were reproduced in our model. Then, the structural optimization was performed using the loose convergence criterion (while fulfilling the preset material project relaxation option) for the generated material candidates, and the formation energy of each was determined (Fig. 2). The formation energies are defined as follows:

1 *E*_f,MgH_ = (*n*_Mg_ × *E*_Mg(solid)_ − *n*_H_ × (*E*_H2(gas)_/2))/*n*_tot_

where *E*_f,MgH_, *E*_Mg(solid)_, and *E*_H2(gas)_ are the total energy of the optimized generated MgH crystal structures, the energy per atom of solid-state pure Mg, and the energy per molecule for H_2_ gas, respectively. *n*_Mg_, *n*_H_, and *n*_tot_ represent the number of elements of Mg, H, and total, respectively.

Fig. 2 shows the phase diagram of the generated Mg_*x*_H_1−*x*_ (the same as that of Mg_*x*_H_*y*_ but with a representation as a proportion of the elements in Mg), which plots the formation energies of all generated structures. In Fig. 2, the red triangles located below the level of zero formation energy indicate the selected structures that primarily satisfy thermodynamic stability. For these selected materials, additional strict criteria for the structural optimization and phonon calculations were included. Consequently, three structures of 2D magnesium hydride monolayers were filtered out: two MgH_2_ with *P*3̄*m*1 (164) and *P*4̄*m*2 (115) space groups and one Mg_2_H_2_ with a *P*1 (2) space group. For brevity, MgH_2_ with *P*3̄*m*1 and *P*4̄*m*2 space groups and Mg_2_H_2_ with a *P*1 space group are referred to as I-phase MgH_2_, II-phase MgH_2_, and III-phase Mg_2_H_2_, respectively.

**Fig. 3:**
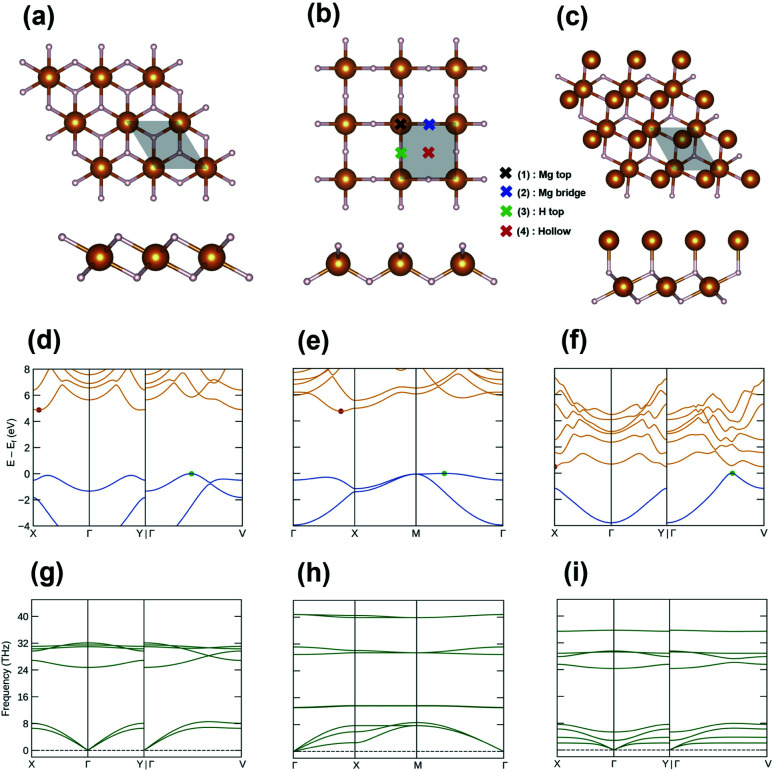
The 2 × 2 supercell of the explored structures: (a) I-phase MgH_2_, (b) II-phase MgH_2_, and (c) III-phase Mg_2_H_2_. The electronic band structures of (d) I-phase MgH_2_, (e) II-phase MgH_2_, and (f) III-phase Mg_2_H_2_. The phonon band structures of (g) I-phase MgH_2_, (h) II-phase MgH_2_, and (i) III-phase Mg_2_H_2_. All band structures were plotted using the Sumo package.^41^

Fig. 3(a)–(c) present the top and side views of the optimized geometries of the discovered structures, and their structural details are summarized in Table 1. The lattice parameters of I-phase MgH_2_ are *a* = *b* = 3.001 Å, *α* = 89.9°, *β* = 89.6°, and *γ* = 119.9°, whereas the parameters of II-phase MgH_2_ are *a* = *b* = 3.198 Å and *α* = *β* = *γ* = 90°. I-phase Mg_2_H_2_ has parameters of *a* = *b* = 3.083 Å, *α* = 87.4°, *β* = 101.3°, and *γ* = 120°. In Fig. 3(d)–(f), the electronic band structures of each nanosheet are plotted with the edges of the valence and conduction bands marked in green and red dots, respectively. I-phase MgH_2_ has an indirect bandgap of 4.88 eV (Fig. 3(d)), II-phase MgH_2_ has an indirect bandgap of 4.74 eV (Fig. 3(e)), and I-phase Mg_2_H_2_ has an indirect bandgap of 0.50 eV (Fig. 3(f)). As can be seen in Fig. 3(g)–(i), these Mg_*x*_H_*y*_ structures are stable depending on their phonon band structures with a nonimaginary frequency.

**Table 1:** The space group, lattice parameters (a, *α*, *β*, and *γ*), formation energy (*E*_f_), and dehydrogenation enthalpies (Δ*H*_d_) of the crystal structures identified using the generative model

Name	Space group	*a* (Å)	*α* (°)	*β* (°)	*γ* (°)	*E*_f_ (eV)	Δ*H*_d_ (eV)
I-phase MgH_2_	*P*3̄*m*1	3.001	89.9	89.6	119.9	−0.130	0.41
II-phase MgH_2_	*P*4̄*m*2	3.198	90.0	90.0	90.0	−0.01	0.05
III-phase Mg_2_H_2_	*P*1	3.083	87.4	101.3	120	−0.03	0.13

The dehydrogenation enthalpy for each compound (Δ*H*_d_ in Table 1) was also calculated. All dehydrogenation enthalpies were calculated with a zero-point energy correction. The lowest value is shown by the II-phase MgH_2_ at 0.05 eV, whereas the values for the I-phase MgH_2_ and III-phase Mg_2_H_2_ are 0.41 and 0.13 eV, respectively. These values are relatively lower than the value of *α*-phase bulk MgH_2_ (0.80 eV).^42^ The lower dehydrogenation enthalpy facilitates easier removal of hydrogen atoms from Mg–H MHs. Therefore, 2D Mg–H MHs were expected to release H_2_ gas at a lower temperature than the *α*-phase bulk MgH_2_.

The I-phase MgH_2_ structure was discovered previously using a substitution-based crystal-prediction model,^27^ whereas III-phase Mg_2_H_2_ possesses an asymmetric structure in which one side is covered with a metal (a polymorph of Mg-decorated I-phase MgH_2_). However, II-phase MgH_2_ has a previously unproposed symmetrical structure with hydrogen covering both of its sides.^27^ So, this paper focuses on the analysis of II-phase MgH_2_. According to the available data from the Materials Project webpage (https://www.materialsproject.org), the most stable structure for MgH_2_ at 0 K is a tetragonal phase MgH_2_ (ID: mp-23710) with 6 atoms in the unit cell and with the symmetry of *P*4_2_/*mnm* (136) (named *α*-phase MgH_2_), Fig. S1(a).† On the other hand, the layered bulk structure of the 2D II-phase of MgH_2_ with a tetragonal symmetry of *P*4_2_/*nmc* (137), is only 0.038 eV per atom less stable than the *α*-phase, Fig. S1(b).† So the slight energy difference of 0.038 eV per atom could be readily compensated for by the energetic fluctuation in a thermally excited statistical system.

To investigate the thermal stability of MgH_2_, (note: MgH_2_ used without a phase modifier essentially means 2D II-phase MgH_2_) AIMD simulations were performed at 300 K up to 6 ps. Top- and side-view photographs of the structures at 0, 3, and 6 ps, respectively, are provided in Fig. S2(a), (b), and (c).† No distortions or defects were identified in the initial pristine structure, which indicates the thermodynamic stability of MgH_2_ under room temperature conditions.

Therefore, it is confirmed that the approach presented herein, which combines a deep learning-based crystal generative model with DFT calculations, can identify unrevealed 2D MgH_2_. Solid crystals can be defined by unit cells, atomic coordinates (structural motifs), and types of elements, although the crystal representation used in this study did not contain information about the latter. Although this model did not generate a new structural motif that was absent in the training data, it was successful in generating the unrevealed structures that were omitted from the classical substitution-based method. With the future development of AI technology in the field of materials, the crystal representation containing information about types of elements and structural motifs will facilitate more effective explorations of new materials.

### 3.2 Decoration of Li atoms on the surface of MgH_2_ and the adsorption of H_2_ molecules

Next, we considered the transition metal (TM) doped MgH_2_. Since it has already been investigated that TM-doped MgH_2_ (α-phase MgH_2_) materials have improved hydrogen adsorption and cycle performance,^43^ TM doping was expected to help improve performances in our 2D MgH_2_ structure. Furthermore, thanks to the characteristics of the 2D layered structure, the doped TMs were expected to serve as nuclei to hold additional H_2_ molecules. Among TMs, such as Al, Ca, K, Li, and Na metals, Li metal was the most stable when doped into the 2D MgH_2_ structure. So, the Li atoms on the surface of MgH_2_ (Li–MgH_2_) were considered to examine the hydrogen storage properties of a single-layer MgH_2_ surface. As shown in Fig. 3(b), four different highly symmetric sites (Mg top, H top, Mg bridge, and hollow) were selected to determine the most stable position. The most stable site for adsorbed Li atoms was found to be located at the site of the Mg bridge by −0.94 eV per unit cell. The binding energy of each Li atom on the MgH_2_ – (2 × 2) supercell is defined as follows:

2 *E*_b_ = *E*_tot_ − *E*_MgH2_ − *E*_Li_

where *E*_tot_, *E*_MgH2_, and *E*_Li_ are the total energy of Li-adsorbed MgH_2_ (Li–MgH_2_), pristine MgH_2_, and the energy for the isolated Li atom, respectively. Table 2 summarizes the binding energies.

**Table 2:** Binding energy of the Li atom on *P*4̄*m*2 MgH_2_

Binding site	*E*_bind,Li_ (eV)
Mg top	−0.412
Mg bridge	−0.924
H top	−0.293
Hollow	−0.731

To clarify the mechanism for H_2_–host interactions, this section considers the adsorption behavior of H_2_ molecules on a single Li-atom-decorated MgH_2_ surface. The adsorption energies are defined as follows:

3 *E*_ad_ = (*E*_tot_ − *E*_Li−MgH2_ − *n*_H2_ × *E*_H2_)/*n*_H2_

where *E*_tot_, *E*_MgH_2__, and *E*_Li–MgH2_ are the total energy of H2 – adsorbed Li–MgH_2_, pristine Li–MgH_2_, and energy for the H_2_ molecules in a vacuum, respectively. *n*_H2_ is the number of H_2_ molecules.

**Fig. 4:**
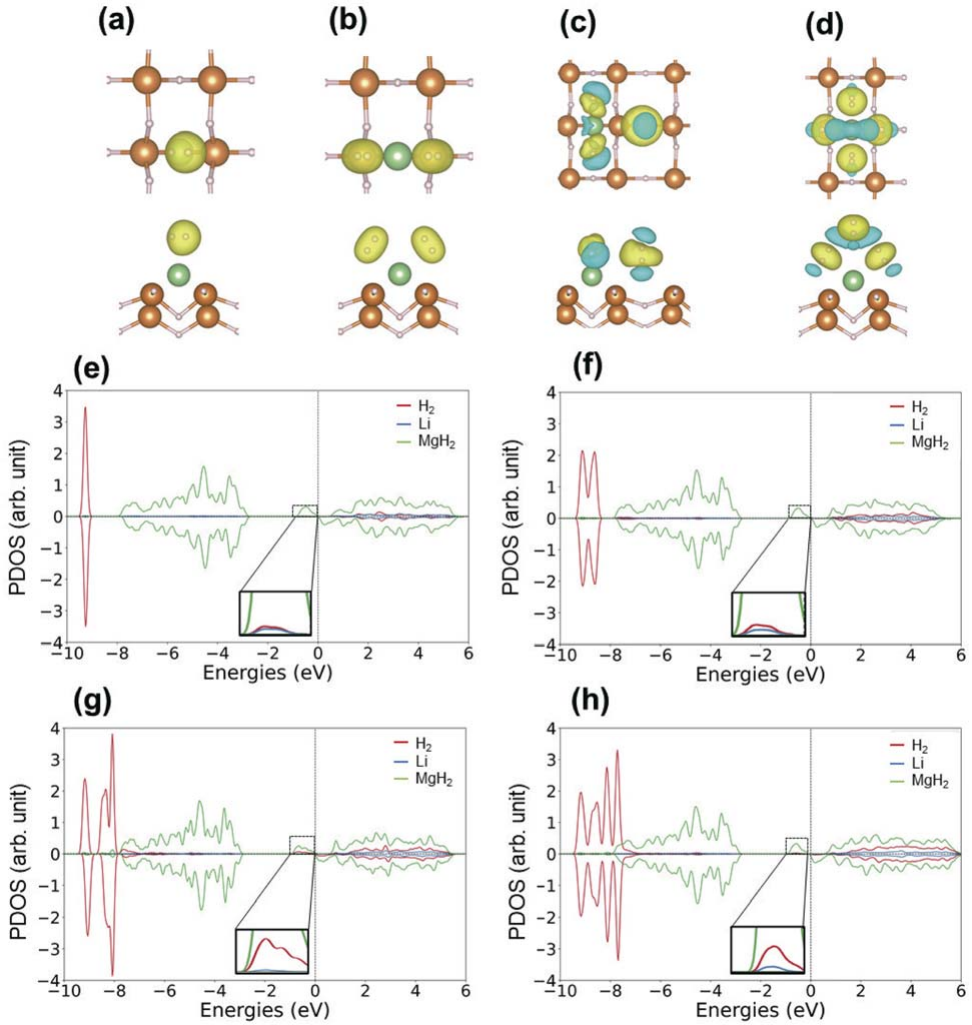
Charge density difference plot of the Li-decorated MgH_2_ system with the adsorption of (a) one H_2_, (b) two H_2_, (c) three H_2_, and (d) four H_2_. The partial density of states (PDOS) of (e) one, (f) two, (g) three, and (h) four H_2_ adsorbed Li–MgH_2_.

H_2_ molecules were adsorbed on the surface of Li–MgH_2_. The average adsorption energies, distances between the Li atoms and hydrogen molecules (*d*_Li–H_), and bond length of the adsorbed H_2_ molecules (*d*_H–H_) as a function of the number of adsorbed H_2_ molecules are summarized in Table 3. The H_2_ bond length is elongated as the number of adsorbed H_2_ molecules increases due to the weakening of the interior H–H bond in the H_2_ molecules resulting from the polarization caused by Li atoms. Multiple potential configurations were considered for the first H_2_ molecule with H_2_ at different angles to the MgH_2_ plane. The optimized ground state structure with an *E*_ad_ of −0.104 eV and a corresponding *d*_H–H_ of 0.754 Å is shown in Fig. 4(a). In this figure, the electrons can be seen to accumulate in H_2_ close to the Li side, whereas the other side demonstrates the contribution to adsorption by the polarization mechanism. This polarization weakens the H–H bond with an elongated *d*_H–H_ of 0.754 Å and aids in subsequent H_2_ adsorption *via* an induction force. To image the process of H_2_ adsorption, additional H_2_ was added in a stepwise manner. As shown in Fig. 4(b), when the second H_2_ approaches the host, H_2_–H_2_ repulsive interactions cause the first one to move to Mg. Here the binding energy per H_2_ molecule is −0.105 eV, and the average H_2_ bond length (*d*_H–H_) is increased from 0.755 to 0.763 Å, as indicated in Table 3. The optimized structure of three H_2_ molecules adsorbed onto Li–MgH_2_ is illustrated in Fig. 4(c). When the third H_2_ is added, the *E*_ad_ increases to −0.111 eV and the average H_2_ bond length increases to 0.763 Å. Then, as shown in Fig. 4(d), the fourth H_2_ is added with an *E*_ad_ of −0.082 eV and a preference to be located opposite to the third one. However, the adsorption behavior of the fourth H_2_ does not follow the same trend; its *E*_ad_ decreases further to −0.082 eV, and the *d*_H–H_ value is reduced further from 0.763 to 0.754 Å. As shown in Fig. 4(c) and (d), in accordance with the adsorption results, there are significantly lower charge-density differences on the third (fourth) H_2_ molecule than on the first and second ones. Thus, up to four hydrogen molecules can exist on one Li-decorated MgH_2_ structure with an adequate adsorption energy (*E*_ad_) of −0.082 eV. The theoretical gravimetric hydrogen density (*G*_theor_) can be estimated using *G*_theor_ = *M*_H2_/(*M*_host_ + *M*_H2_), where *M*_host_ and *M*_H2_ indicate the quality of the host material and the H_2_ molecules, respectively. Consequently, the Li–MgH_2_ (4H_2_) system can be expected to produce a remarkable *G*_theor_ of 6 wt%.

**Table 3:** The calculated adsorption energy (*E*_ad_) values of H_2_ on Li–MgH_2_. *d*_Li–H2_ and *d*_H–H_ represent the average distance of Li to the H_2_ and H–H bond lengths, respectively

Number of H_2_ molecules	*E*_ad,H2_ (eV)	*d*_Li–H2_ (Å)	*d*_H–H_ (Å)
1	−0.104	2.113	0.754
2	−0.105	2.244	0.755
3	−0.111	2.556	0.763
4	−0.082	2.911	0.754

Next, the hydrogen-binding mechanism was clarified by investigating the electronic properties of the surface of the H_2_/Li–MgH_2_ structure. Fig. 4(e) and (f) show the PDOS of Li–MgH_2_ with one and two H_2_ molecules. With a single H_2_ molecule, the states of H and Li are hybridized around the peaks at −9.4 and −0.4 eV in Fig. 4(e). As shown in Fig. 4(f), for two H_2_ molecules, the hybridized peak at −9.4 eV divides into two at −9.6 and −8.6 eV, respectively. With the addition of the third and fourth H_2_ molecules, the hybridization peak at −9.4 eV splits into three and four peaks according to the number of H_2_ molecules, as shown in Fig. 4(g) and (h). In the system with three adsorbed H_2_ molecules (Fig. 4(g)), the split hybridized peaks are located at −9.6, −8.6, and −8.0 eV, respectively. As shown in Fig. 4(g), (four H_2_ molecules), the split hybridized peaks are located at −9.5, −8.5, −8.1, and −7.8 eV, respectively. The division of the hybridization peaks reduces the intensity of the individual peaks, which indicates a reduced bond strength between the H_2_ molecule and host material in accordance with the previous analysis.

Consequently, four additional H_2_ molecules could be stored by Li-decorated 2D MgH_2_ with an *E*_ad_ of −0.105 eV and a *G*_theor_ of 6 wt%. Considering that the host material in this study is 2D MH, which contains a chemisorbed hydrogen source, it can be said to possess additional hydrogen storage capacity, which makes it competitive against other Li-decorated 2D hydrogen host materials, including h-BN (∼6 wt%)^23^ and borophene (6.80–11.49 wt%).^44,45^ Therefore, the Li-decorated system of a 2D MgH_2_ sheet with a *P*4̄*m*2 space group is anticipated to be a viable medium for substantial hydrogen storage.

## 4 Conclusions

This study successfully demonstrated that a deep learning-based crystal structure-prediction model can generate reliable 2D materials with the potential for effective and substantial hydrogen storage. The GAN model in this study effectively explored the structural space of the 2D material and identified an unknown structure, which has not been proposed previously using classical substitution-based methods. The 2D MgH_2_ material with a *P*4̄*m*2 space group was confirmed to be a thermally stable structure with the potential for significant hydrogen storage. The hydrogen storage properties of the pristine and Li-decorated *P*4̄*m*2 MgH_2_ monolayer were investigated using the DFT method, and it was revealed that pristine MgH_2_ can act as a source of chemisorbed hydrogen with a dehydrogenation enthalpy of 0.05 eV. Additionally, four additional H_2_ molecules are capable of being physisorbed onto the surface of 2D Li-decorated MgH_2_ to produce efficient hydrogen storage materials with an average H_2_ molecular adsorption energy of −0.105 eV and a gravimetric hydrogen capacity of 6 wt%.

This study's findings indicate that the combined approach of AI and DFT calculations can be used to predict 2D crystal structures efficiently; consequently, 2D *P*4̄*m*2 MgH_2_ is proposed as a novel hydrogen storage medium. It is anticipated that this study's findings will accelerate the development of hydrogen storage materials and other desired functional materials both theoretically and experimentally.

## Code and data availability

As mentioned, all DFT calculations were performed using the VASP package. Furthermore, the original code of the generative deep learning model was obtained from https://github.com/kaist-amsg/Composition-Conditioned-Crystal-GAN. We modified the code to suit the purpose of our study. The code and data used in this study are provided along with the ESI† in the code_data.zip.

## Author contributions

Junho Lee: conceptualization, data curation, investigation, methodology, visualization, validation, writing – original draft, writing – review & editing. Dongchul sung: methodology, validation, supervision, writing – review & editing. You Kyoung Chung: validation, writing review & editing. Seon Bin Song: validation, visualization. Joonsuk Huh: project administration, supervision, funding acquistion, writing – review & editing.

## Conflicts of interest

There are no conflicts to declare.

## Supplementary Material

NA-004-D1NA00862E-s001
