# Supplementary material for: Unveiling two-dimensional magnesium hydride as a hydrogen storage material via a generative adversarial network
Source: Nanoscale Adv. 2022 Apr 8;4(10):2332–8. doi: 10.1039/d1na00862e (PMC9418009; doi:10.1039/d1na00862e)
Supplement: NA-004-D1NA00862E-s001 [file NA-004-D1NA00862E-s001.pdf]

Electronic Supplementary Material (ESI) for Nanoscale Advances.
This journal is © The Royal Society of Chemistry 2022

Supplementary Information

Unveiling two-dimensional magnesium hydride for hydrogen storage material via generative adversarial network

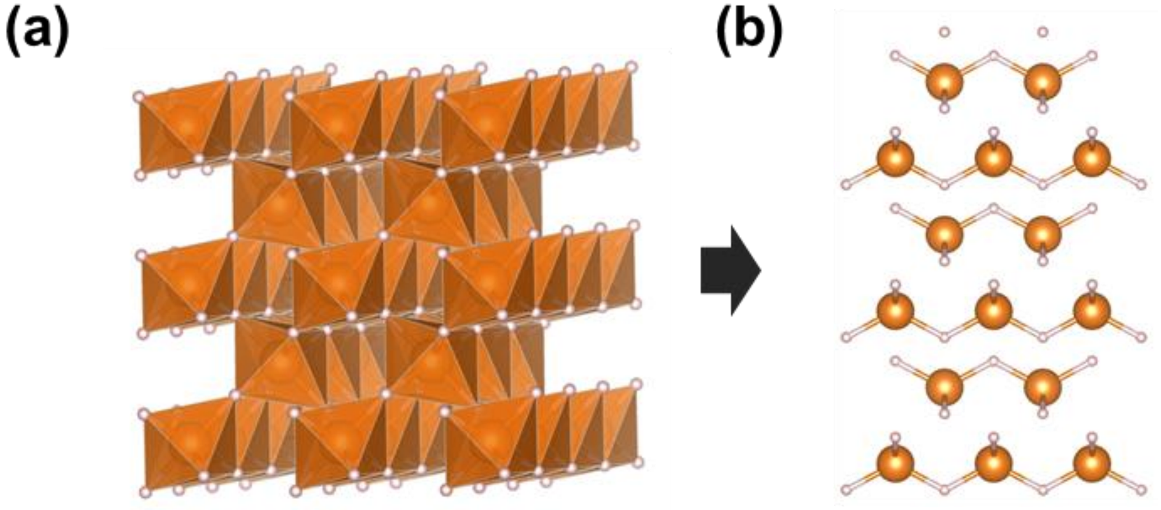

Figure S1. Two phases of MgH_2, (a) the tetragonal MgH_2 with symmetry of $P4_2/mnm$, *α*-phase and (b) the layered bulk structure of 2D II-phase, $P4_2/nmc$.

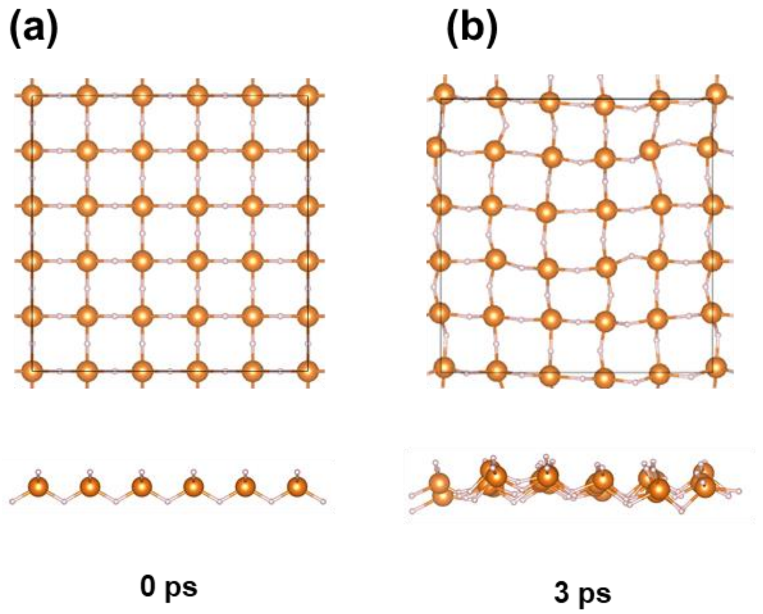

Figure S2. Snapshots (top and side views) of 5 × 5 × 1 supercell of 2D *P-4m2* MgH_2 at the (a) 0 ps (b) 3 ps and (c) 6 ps at 300K
